# A new species of the genus *Diploderma* (Reptilia, Squamata, Agamidae) from Zayü County, Xizang Autonomous Region, China

**DOI:** 10.3897/zookeys.1296.198381

**Published:** 2026-11-04

**Authors:** Bo Cai, Fengjing Liu, Ayimo Budian, Zhongyi Yao, Boyang Wu, Jianping Jiang, Xiaobo Luo, Keji Guo

**Affiliations:** 1 Chengdu Institute of Biology, Chinese Academy of Sciences, Chengdu 610213, China Chengdu Institute of Biology, Chinese Academy of Sciences Chengdu China; 2 Jiangsu Key Laboratory for the Biodiversity Conservation and Sustainable Utilization in the Middle and Lower Reaches of the Yangtze River Basin, College of Life Sciences, Nanjing Normal University, Nanjing 210023, China Jiangsu Key Laboratory for the Biodiversity Conservation and Sustainable Utilization in the Middle and Lower Reaches of the Yangtze River Basin, College of Life Sciences, Nanjing Normal University Nanjing China; 3 Sichuan Academy of Natural Resource Sciences, Chengdu 610041, China Sichuan Academy of Natural Resource Sciences Chengdu China; 4 Kunming Institute of Zoology, Chinese Academy of Sciences, Kunming 65020, China Kunming Institute of Zoology, Chinese Academy of Sciences Kunming China; 5 College of Life-science and Technology, Central South University of Forestry and Technology, Changsha 410004, China College of Life-science and Technology, Central South University of Forestry and Technology Changsha China; 6 Central South Inventory and Planning Institute of National Forestry and Grassland Administration, Changsha 410014, China Central South Inventory and Planning Institute of National Forestry and Grassland Administration Changsha China

**Keywords:** *Diploderma gulaense* sp. nov., dry valley, geographical isolation, Hengduan mountains, Mountain Lizard, Qinghai-Tibet Plateau, Salween River

## Abstract

A new species of the agamid genus *Diploderma* Hallowell, 1861 is described from Gula Township, Zayü County, Xizang Autonomous Region (Tibet), China, based on integrated morphological and molecular evidence. Phylogenetic analyses of mitochondrial ND2 genes reveal that the new species *Diploderma gulaense* Cai, Yao, Wu & Guo, **sp. nov**. forms a well-supported clade with *D. laeviventre*, *D. yangi*, and *D. slowinskii*, endemic to the Nu River valley. It is sister to *D. laeviventre* but differs from it and other congeners by a combination of morphological characteristics: moderate body size (SVL 53.1–63.4 mm in males, 54.8–70.2 mm in females); relatively long tail (TAL/SVL 2.07–2.38 in males, 1.90–2.26 in females); tympanum concealed; ventral body scales strongly keeled; transverse gular fold present; cyan gular spot in both sexes; pale cyan dorsolateral stripes in males; and tongue pale flesh colored. The new species is currently known only from the Ranbuqu valley, a dry-warm valley along a tributary of the middle Nu River, at elevations between 2,332 and 3,300 m. Its restricted distribution and ongoing habitat degradation suggest a preliminary IUCN assessment of Vulnerable (VU). The discovery of *Diploderma gulaense* Cai, Yao, Wu & Guo, **sp. nov**. further highlights the role of river-valley systems in driving allopatric speciation within the genus and underscores the underestimated diversity of *Diploderma* in the Hengduan Mountains.

## Introduction

The agamid genus *Diploderma* Hallowell, 1861, represents one of the most species-rich genera among reptiles in China (Liu et al. 2025; Cai 2026). The diversity of the genus is concentrated in mainland China, particularly in the Hengduan mountains, a biodiversity hotspot recognized for its exceptional levels of endemism and habitat diversity (Myers et al. 2000). This mountainous region, characterized by a series of parallel north-south oriented river valleys, has facilitated both vicariance and in-situ speciation, making it the primary centre of diversification for the genus (Liu et al. 2023, 2025).

**Figure 1.**
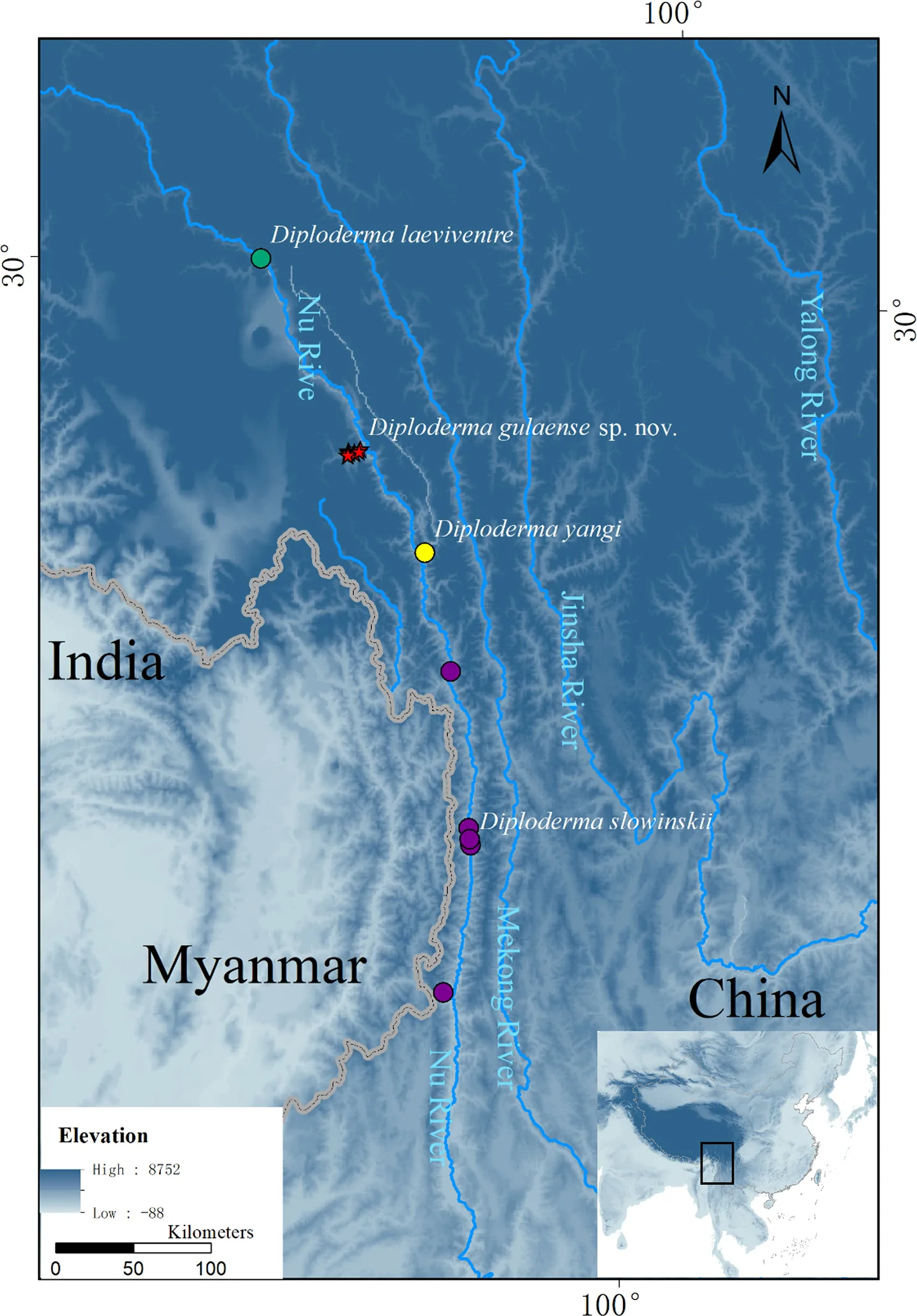
Map showing the locations of *Diploderma* specimens in the Nu River Valley (= upper Salween River Valley). The red stars are *Diploderma gulaense* sp. nov., the green circles represent *D. laeviventre*, yellow circles *D. yangi*, and purple circles *D. slowinskii* from top to bottom.

Within the Hengduan mountains, the Nu River (also known as the Salween River) is a particularly significant biogeographic feature. The river is traditionally divided into three sections: the upper reach from its headwaters on the Tibetan Plateau to Jiayuqiao in eastern Xizang, the middle reach, commonly known as the Nu Valley, from Jiayuqiao southward to Lushui in Yunnan Province, and the lower reach from Lushui downstream to the river mouth in the Andaman Sea (Liu et al. 2008). The valley exhibits a complex geologic and climatic gradient, ranging from high-altitude, cold-dry habitats in the north to warmer, more temperate zones in the south (Pan et al. 2006; Du et al. 2009). To date, several *Diploderma* species have been recorded from the Nu River drainage, including *D. slowinskii* (Rao, Vindum, Ma, Fu & Wilkinson, 2017) (Rao et al. 2017) from the middle reaches in Yunnan, and *D. laeviventre* (Wang, Jiang, Siler & Che, 2016) (Wang et al. 2016) and the recently described *D. yangi* Wang, Zhang & Li, 2022 (Wang et al. 2022) from Xizang (Fig. 1). Such findings underscore that the intricate landscape of the middle reach Nu River valley supports a greater diversity of lizards than previously recognized.

In August 2019 and June 2023, a series of specimens of *Diploderma* were collected during field surveys in Zayü County, Xizang Autonomous Region, China, an area situated within the middle reach of Nu River drainage. Subsequent molecular phylogenetic analysis and detailed morphological comparisons revealed that these specimens represent a lineage distinct from all known congeners. In this study, we describe this population as a new species and provide diagnostic characters that differentiate it from its closest relatives.

## Materials and methods

### Sampling

A total of 13 specimens were collected in Gula Township, Zayü County, Xizang Autonomous Region, China. After taking photographs of these live animals to document their color patterns, they were euthanized with 75% ethanol. Liver tissue samples were taken and preserved in 95% ethanol for molecular analyses. All specimens were fixed in 75% ethanol and deposited at the Museum of Herpetology, Chengdu Institute of Biology (**CIB**), Chinese Academy of Sciences (**CAS**).

### DNA extraction and sequences of ND2

Total genomic DNA was extracted from liver tissue samples of 9 specimens collected in this study using the QIAamp DNA Mini Kit (QIAGEN, Hilden, Germany), following the manufacturer’s instructions. A fragment of the mitochondrial NADH dehydrogenase subunit 2 (ND2) gene was amplified and sequenced. The primer pair Jap_70F (5'-CCACCAAACAACTACACCTA-3') and Jap_1559R (5'-GGATTAATGCCCTCTGGATT-3') was adopted from Wang et al. (2019). PCR amplification and sequencing procedures followed Liu et al. (2023).

### Molecular phylogenetic analyses

The newly obtained sequences were assembled and edited based on forward and reverse reads using SeqMan 7.1.0.44 (Skwor 2012) in the DNASTAR 7.1 software package (DNAStar Inc., Madison, WI, USA). Nine newly generated ND2 sequences were obtained in this study, and an additional 98 ND2 sequences representing 47 recognized species of *Diploderma* were downloaded from GenBank for phylogenetic analyses (Table 1). In addition, the sequence of *Pseudocalotes brevipes* (AF128502) was used as the outgroup.

**Table 1.** GenBank accession numbers for the sequences used in this study.

	**Species**	**Voucher**	**Locality**	**Accession**
1	*Diploderma gulaense* sp. nov.	CIB-CB23CY01	Zayü, Xizang, China	PZ412418
		CIB-CB23CY02	Zayü, Xizang, China	PZ412419
		CIB-CB23CY03	Zayü, Xizang, China	PZ412420
		CIB-07564	Zayü, Xizang, China	PZ412425
		CIB-07565	Zayü, Xizang, China	PZ412426
		CIB-07566	Zayü, Xizang, China	PZ412421
		CIB-07567	Zayü, Xizang, China	PZ412422
		CIB-07568	Zayü, Xizang, China	PZ412423
		CIB-07569	Zayü, Xizang, China	PZ412424
2	*D. angustelinea*	KIZ029704	Muli, Sichuan, China	MT577930
		KIZ029705	Muli, Sichuan, China	MT577924
3	*D. aorun*	KIZ032733	Benzilan, Yunnan, China	MT577938
		KIZ032734	Benzilan, Yunnan, China	MT577939
4	*D. batangense*	KIZ09404	Zhubalong, Xizang, China	MK001412
		KIZ019276	Batang, Sichuan, China	MK001413
5	*D. bifluviale*	CB23JC04	Barkam, Sichuan, China	PV833285
		CB23JC05	Barkam, Sichuan, China	PV833286
6	*D. brevicauda*	KIZ044305	Lijiang, Yunnan, China	MW506021
		KIZ044306	Lijiang, Yunnan, China	MW506022
7	*D. bowoense*	KIZ044757	Muli, Sichuan, China	MW506020
		KIZ044758	Muli, Sichuan, China	MW506019
8	*D. brevipes*	NMNS19607	Taiwan, China	MK001429
		NMNS19608	Taiwan, China	MK001430
9	*D. chapaense*	KIZ034923	Lvchun, Yunnan, China	MG214263
		ZMMUNAP-01911	Chapa, Vietnam	MG214262
10	*D. danbaense*	KIZ2022048	Danba, Sichuan, China	OQ378180
		KIZ2022049	Danba, Sichuan, China	OQ378181
11	*D. daduense*	CB2021227	Hanyuan, Sichuan, China	PP539949
		CB2021228	Hanyuan, Sichuan, China	PP539950
12	*D. daochengense*	20210905	Muli, Sichuan, China	OP595620
		DC001	Daocheng, Sichuan, China	OP595621
13	*D. donglangense*	KIZ2022057	Muli, Sichuan, China	OQ378185
		KIZ2022058	Muli, Sichuan, China	OQ378186
14	*D. drukdaypo*	KIZ027627	Jinduo, Xizang, China	MT577950
		KIZ027628	Zhuka, Xizang, China	MT577952
15	*D. dymondi*	KIZ040639	Dongchuan, Yunnan, China	MK001422
		KIZ040640	Dongchuan, Yunnan, China	MK001423
16	*D. fasciatum*	SYS r002175	Wuming, Guangxi, China	OM055809
		KIZ040192	Daweishan, Yunnan, China	OM055800
17	*D. flaviceps*	KIZ01851	Luding, Sichuan, China	MK001416
		KIZ01852	Luding, Sichuan, China	MK001417
18	*D. flavilabre*	KIZ032692	Baiyu, Sichuan, China	MT577916
		KIZ032694	Baiyu, Sichuan, China	MT577917
19	*D. formosgulae*	KIZ044420	Deqin, Yunnan, China	MW506024
		KIZ044421	Deqin, Yunnan, China	MW506025
20	*D. iadinum*	KIZ027697	Yunling, Yunnan, China	MT577956
		KIZ027702	Yunling, Yunnan, China	MT577957
21	*D. jiulongense*	KIZ2022086	Jiulong, Sichuan, China	OQ378190
		KIZ2022087	Jiulong, Sichuan, China	OQ378191
22	*D. kangdingense*	20210916	Kangding, Sichuan, China	OP595625
		20210917	Kangding, Sichuan, China	OP595626
23	*D. laeviventre*	KIZ014037	Baxoi, Xizang, China	MK001407
		KIZ027691	Baxoi, Xizang, China	MT577892
		KIZ027692	Baxoi, Xizang, China	MT577893
24	*D. limingense*	KIZ2022014	Yulong, Yunnan, China	OP428782
		KIZ2022015	Yulong, Yunnan, China	OP428783
25	*D. luei*	NMNS19604	Taiwan, China	MK001433
		NMNS19605	Taiwan, China	MK001434
26	*D. makii*	NMNS19609	Taiwan, China	MK001431
		NMNH19610	Taiwan, China	MK001432
27	*D. menghaiense*	KIZ L0030	Menghai, Yunnan, China	MT598655
		KIZ L0031	Menghai, Yunnan, China	MT598656
28	*D. micangshanense*	KIZ032801	Shiyan, Hubei, China	MK578665
		KIZ023231	Xixia, Henan, China	MK578664
29	*D. nangunhe*	KIZ2024100	Gengma, Yunnan, China	PQ629458
		KIZ2020012	Gengma, Yunnan, China	PQ629459
30	*D. panchi*	KIZ032715	Yajiang, Sichuan, China	MT577946
		KIZ032716	Yajiang, Sichuan, China	MT577944
31	*D. panlong*	KIZ040137	Miansha, Sichuan, China	MT577906
		KIZ040138	Miansha, Sichuan, China	MT577907
32	*D. polygonatum*	NMNS19598	Taiwan, China	MK001427
		NMNS19599	Taiwan, China	MK001428
33	*D. qiaojiaense*	KIZ2021101	Qiaojia, Yunnan, China	PP889934
		KIZ2021102	Qiaojia, Yunnan, China	PP889935
34	*D. qilin*	KIZ028332	Deqin, Yunnan, China	MT577941
		KIZ028333	Deqin, Yunnan, China	MT577942
35	*D. shuoquense*	KIZ2022004	Xiangcheng, Sichuan, China	OP428773
		KIZ2022005	Xiangcheng, Sichuan, China	OP428774
36	*D. slowinskii*	CAS214906	Gongshan, Yunnan, China	MK001405
		CAS214954	Gongshan, Yunnan, China	MK001406
		KIZ027543	Gongshan, Yunnan, China	MT577910
37	*D. splendidum*	KIZ015973	Yichang, Hubei, China	MK001418
		LSUMZ81212	Unknown	AF288230
38	*D. swild*	KIZ034914	Panzhihua, Sichuan, China	MN266299
		KIZ034894	Panzhihua, Sichuan, China	MN266300
39	*D. swinhonis*	NMNS19592	Taiwan, China	MK001419
		NMNS19593	Taiwan, China	MK001420
40	*D. tachengense*	KIZ2022028	Weixi, Yunnan, China	OQ378195
		KIZ2022027	Weixi, Yunnan, China	OQ378196
41	*D. varcoae*	WK-JK 011	Yuxi, Yunnan, China	MT577903
		KIZ026132	Mengzi, Yunnan, China	MK001421
42	*D. vela*	KIZ019299	Quzika, Xizang, China	MK001414
		KIZ034925	Quzika, Xizang, China	MK001415
43	*D. xinlongense*	20210907	Xinlong, Sichuan, China	OP595613
		20210908	Xinlong, Sichuan, China	OP595614
44	*D. yangi*	SWFU005410	Zayü, Xizang, China	OL449603
		SWFU005411	Zayü, Xizang, China	OL449606
		SWFU005412	Zayü, Xizang, China	OL449604
		SWFU005414	Zayü, Xizang, China	OL449605
45	*D. yongshengense*	KIZ2022009	Yongsheng, Yunnan, China	OP428777
		KIZ2022008	Yongsheng, Yunnan, China	OP428778
46	*D. yulongense*	KIZ028291	Hutiaoxia, Yunnan, China	MT577921
		KIZ028292	Hutiaoxia, Yunnan, China	MT577922
47	*D. yunnanense*	CAS242271	Baoshan, Yunnan, China	MK001408
		KIZ040193	Yingjiang, Yunnan, China	MK578658
48	*D. zhaoermii*	KIZ019564	Wenchuan, Sichuan, China	MK001425
		KIZ019565	Wenchuan, Sichuan, China	MK001426
49	*Pseudocalotes brevipes*	MVZ224106	Vinh Phuc, Vietnam	AF128502

Sequences were aligned in MEGA X (Kumar et al. 2018) using ClustalX (Thompson et al. 1997) with default settings. Phylogenetic analyses were conducted in PhyloSuite v. 1.2.3 (Zhang et al. 2020) using MrBayes v. 3.2.7a (Ronquist et al. 2012) for Bayesian inference (BI) and IQ-TREE v. 2.2.0 (Nguyen et al. 2015) for maximum likelihood (ML) analyses. Prior to the phylogenetic analyses, the best evolutionary model was selected based on the Bayesian Information Criterion (BIC) using ModelFinder (Kalyaanamoorthy et al. 2017). Prior to the phylogenetic analyses, the dataset was partitioned by codon position. The best-fit substitution models for each partition were selected based on the BIC using ModelFinder [Model GTR + I + G4]. In the BI analyses, two runs were performed simultaneously with four Markov chains. The chains were run for 10,000,000 generations and sampled every 1000 generations. The first 25% of sampled trees were discarded as burn-in. Convergence was assessed using the diagnostics implemented in MrBayes; minimum ESS values for all model parameters exceeded 3,800 and PSRF values were 1.000. The remaining trees were used to estimate Bayesian posterior probabilities (BPPs). Nodes with BPP values ≥ 0.95 were considered well supported (Huelsenbeck et al. 2001; Wilcox et al. 2002). The ML analysis was conducted in IQ-TREE v. 2.2.0 under the GTR + F + I + G4 model with 1,000 ultrafast bootstrap replicates. Nodes with ultrafast bootstrap support (UFBS) values ≥ 95% were considered well supported (Minh et al. 2013). Finally, uncorrected genetic pairwise distances (*p*-distances) for ND2 were calculated using default parameters in MEGA X (Kumar et al. 2018). Gaps and missing data were treated using partial deletion with a site coverage cutoff of 95%.

### Morphological analyses

Morphological data of recognized *Diploderma* species were obtained from examination of museum specimens deposited in the Chengdu Institute of Biology (**CIB**), as well as from the literature (Wang et al. 2016, 2022; Rao et al. 2017). We are aware that measurements derived from literature may introduce minor bias; therefore, we employed principal component analysis (see below) to visualize morphological differences, and we emphasize characters (e.g., pholidosis counts and color patterns) that are less prone to measurement artefacts.

Measurements were taken primarily following Zhao et al. (1999) and Liu et al. (2024). All measurements, including snout–vent length (**SVL**) and tail length (**TAL**), were taken to the nearest 0.1 mm using a digital caliper. A total of eleven measurements were recorded: **SVL** (distance from the snout tip to the anterior edge of the vent); **TAL** (distance from the anterior edge of the vent to the tip of the tail); head width **(HW)** (distance between the widest points in the temporal region, anterior to the tympanum); head length **(HL)** (distance from the tip of the snout to the right angle of the jaw); head depth **(HD)** (perpendicular distance at the temporal region of the head); snout-eye length **(SEL)** (distance from the tip of the snout to the anterior margin of the eye); length of the tallest nuchal crest **(TNC)** (distance from the base to the tip of the tallest nuchal crest scale); foreleg length **(FLL)** (distance from the armpit to the tip of finger IV, excluding the claw, with the limb straightened); hindleg length **(HLL)** (distance from the groin to the tip of toe IV, excluding the claw, with the limb straightened); trunk length **(TRL)** (distance from the armpit to the groin); and toe IV length **(T4L)** (distance from the tip of toe IV to the base between toes III and IV, excluding the claw).

Definitions of morphological characters and counting methods also mainly followed Zhao et al. (1999) and Liu et al. (2024). The following pholidosis characters were recorded: supralabial scale count **(SL)** (number of enlarged, modified labial scales from the rostral to the corner of the mouth); infralabial scale count **(IL)** (number of enlarged, modified labial scales from the mental to the corner of the mouth); canthus rostralis scale count **(CR)** (number of elongated, overlapping scales from posterosuperior nasal to and including last supraciliary scale along the canthal ridge); nasal–supralabials scale rows **(NSL)** (number of horizontal rows of small scales between the first supralabial and the nasal); suborbital scale rows **(SoR)** (number of longitudinal rows of scales between supralabials and inferior-most edge of orbit circle, excluding fine ciliary scales in the orbit); ventral scale count **(VN)** (number of ventral body scales counted in a straight line along the medial axis between the transverse gular fold and the anterior edge of the cloaca); gular scale count **(GU)** (number of gular scales counted in a straight line along the medial axis, excluding the mental and the transverse gular fold); middorsal crest scale count **(MD)** (number of modified crest scales counted longitudinally from the first nuchal crest to the scale above the cloaca); finger IV subdigital lamellae count **(F4S)** (number of left subdigital lamellae from the base between fingers III and IV to the tip of finger IV, excluding the claw); toe IV subdigital lamellae count **(T4S)** (number of left subdigital lamellae from the base between toes III and IV to the tip of toe IV, excluding the claw).

Coloration descriptions follow the terminology and codes of the RGB (red, green, blue) color model (Cai et al. 2022). Data on coloration and ornamentation were collected from live specimens both in a resting state and under stress, including inner-lip coloration (**ILC**); coloration of the oral cavity (**CO**): background coloration of the anterior roof and sides of the mouth, excluding the posterior palate and deep throat); coloration of the tongue (**CTG**); coloration of the dorsolateral stripes (**CDS**); ventrolateral body coloration (**VLBC**); and ventral body coloration (**VBC**).

Principal component analysis (PCA) was conducted to visualize multivariate morphometric variation among the focal taxon and its closely related congeners based on nine characters: SVL, TAL, HW, HL, SEL, FLL, HLL, T4L, and TRL. Prior to PCA, each variable was centered and scaled to unit variance. PCA was performed using the prcomp function in R v. 4.5.3 (R Core Team 2026) with center = TRUE and scale. = TRUE, and scatterplots were generated using ggplot2 3.4.4 (Wickham 2016). Because sexual dimorphism in morphometric characters has been widely reported in *Diploderma* (Wang et al. 2019; Cai et al. 2022; Liu et al. 2023), males and females were analyzed separately. The PCA was used as a descriptive visualization of morphometric variation rather than as a statistical test of differences among species.

To incorporate both categorical (e.g., color pattern, tuberculation) and numerical characters in a single analytical framework, we also performed a multiple factor analysis (MFA). MFA extends PCA by first computing separate ordinations for each data type, then normalising them so that no single variable set dominates the combined solution. This approach allows categorical traits—often omitted from multivariate analyses—to contribute meaningfully to species discrimination (Grismer 2025). The analysis was conducted using the FactoMineR package (Husson et al. 2017) in R (Suppl. materal 1: table SS1), incorporating both numerical and categorical morphological characters. PERMANOVA was based on the first five MFA dimensions using Euclidean distances, 50,000 permutations, and Bonferroni correction (Oksanen et al. 2020).

## Results

### Phylogenetic results

**Figure 2.**
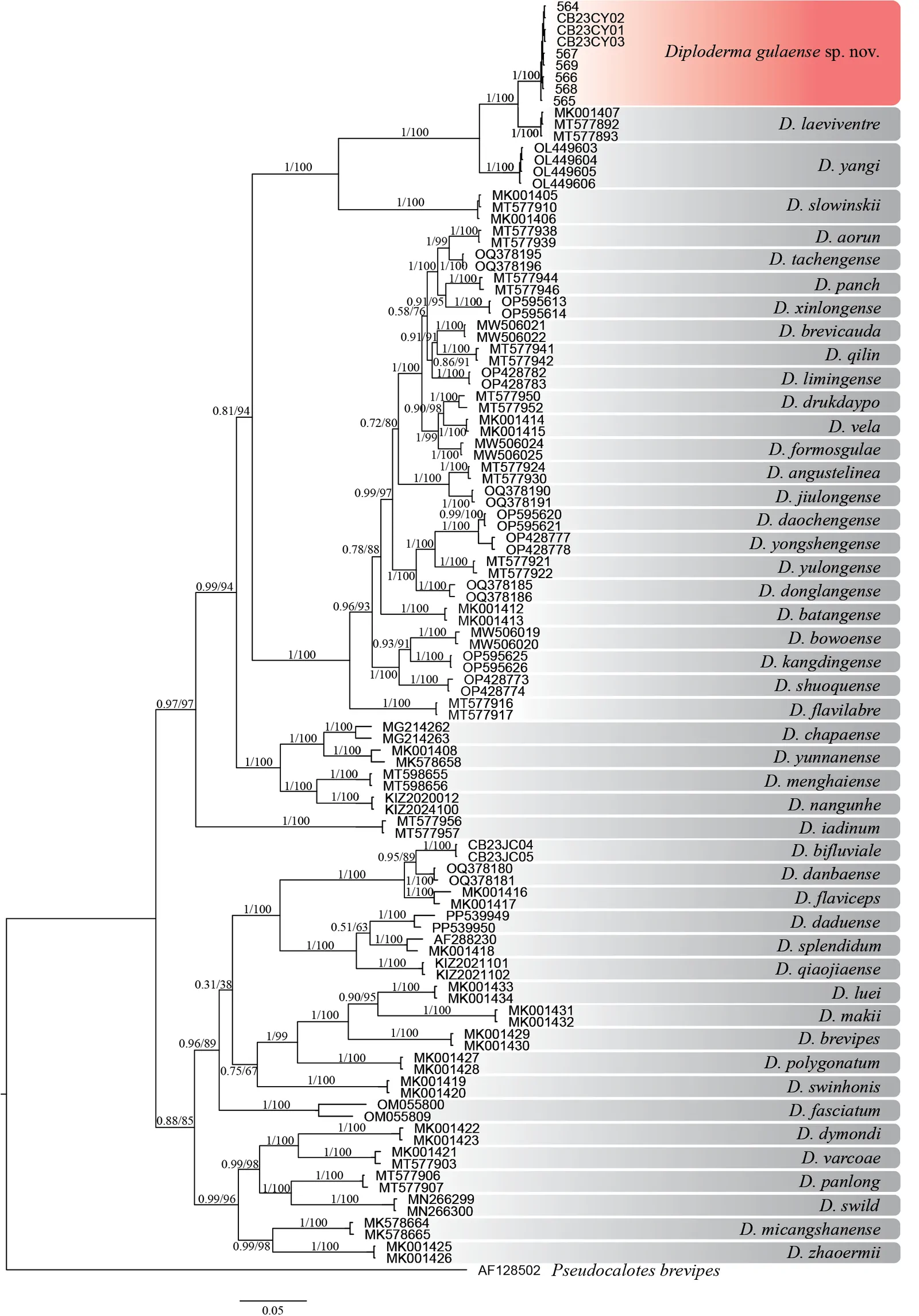
The phylogenetic tree of the genus *Diploderma* inferred from the mitochondrial gene ND2. Bayesian posterior probabilities and ML bootstrap support values are denoted above each node.

The BI and ML analyses produced identical topologies. In both analyses, the specimens from Gula Township were recovered as a distinct and well-supported monophyletic lineage. All samples from the Gula Township population clustered together with strong support (BPP/UFBS = 1/100). The Gula Township lineage was inferred to be the sister taxon of *D. laeviventre*, and this relationship was strongly supported (BPP/UFBS = 1/100). The Gula lineage, together with *D. laeviventre*, *D. yangi*, and *D. slowinskii*, constitutes the Nu River lineage of the genus, in which the Gula population and *D. laeviventre* form a well-supported clade, to which *D. yangi* and *D. slowinskii* are successively related (Fig. 2).

The uncorrected mean genetic distance between the Gula Township lineage and its closest relative, *D. laeviventre*, was 3.06%, which is within the range observed among other recognized *Diploderma* species (Suppl. materal 1: table SS2).

### Morphological results

**Figure 3.**
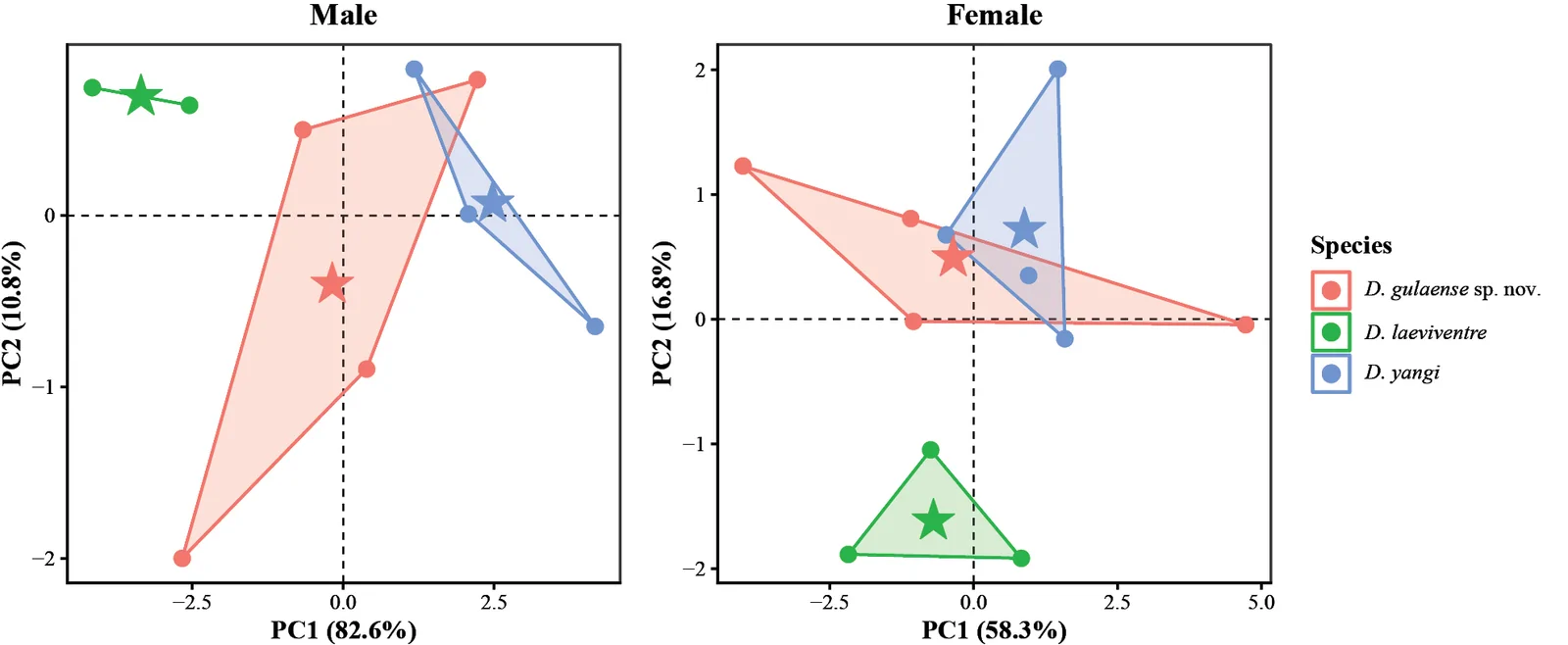
PCA based on nine morphometric characteristics (SVL, TAL, HW, HL, SEL, FLL, HLL, T4L, and TRL) for *Diploderma gulaense* sp. nov., *D. yangi*, and *D. laeviventre*. Numbers inside the brackets indicate the percentages of the total variance explained by each axis.

The PCA revealed morphological differentiation among the Gula Township population, *D. laeviventre*, and *D. yangi* in both sexes. In males, PC1 and PC2 explained 82.59% and 10.81% of the total variance, respectively, together accounting for 93.40% of the variation (Suppl. materal 1: table SS3). In females, PC1 and PC2 explained 58.33% and 16.79% of the total variance, respectively, together accounting for 75.12% of the variation (Suppl. materal 1: table S4). In the male dataset, the morphospace of the Gula Township population showed slight overlap with *D. laeviventre* along PC1 and partial overlap with *D. yangi*. In the female dataset, broader overlap was observed among the three taxa, particularly along PC1. Thus, the PCA did not show complete morphometric separation among the three taxa (Fig. 3). In both sexes, all variables loaded in the same direction on PC1, indicating that this axis largely reflected overall size-related variation. In males, PC2 was influenced mainly by TRL, SEL, and T4L (Suppl. materal 1: table SS3), whereas in females it was influenced mainly by TRL, SEL, and FLL (Suppl. materal 1: table S4).

**Figure 4.**
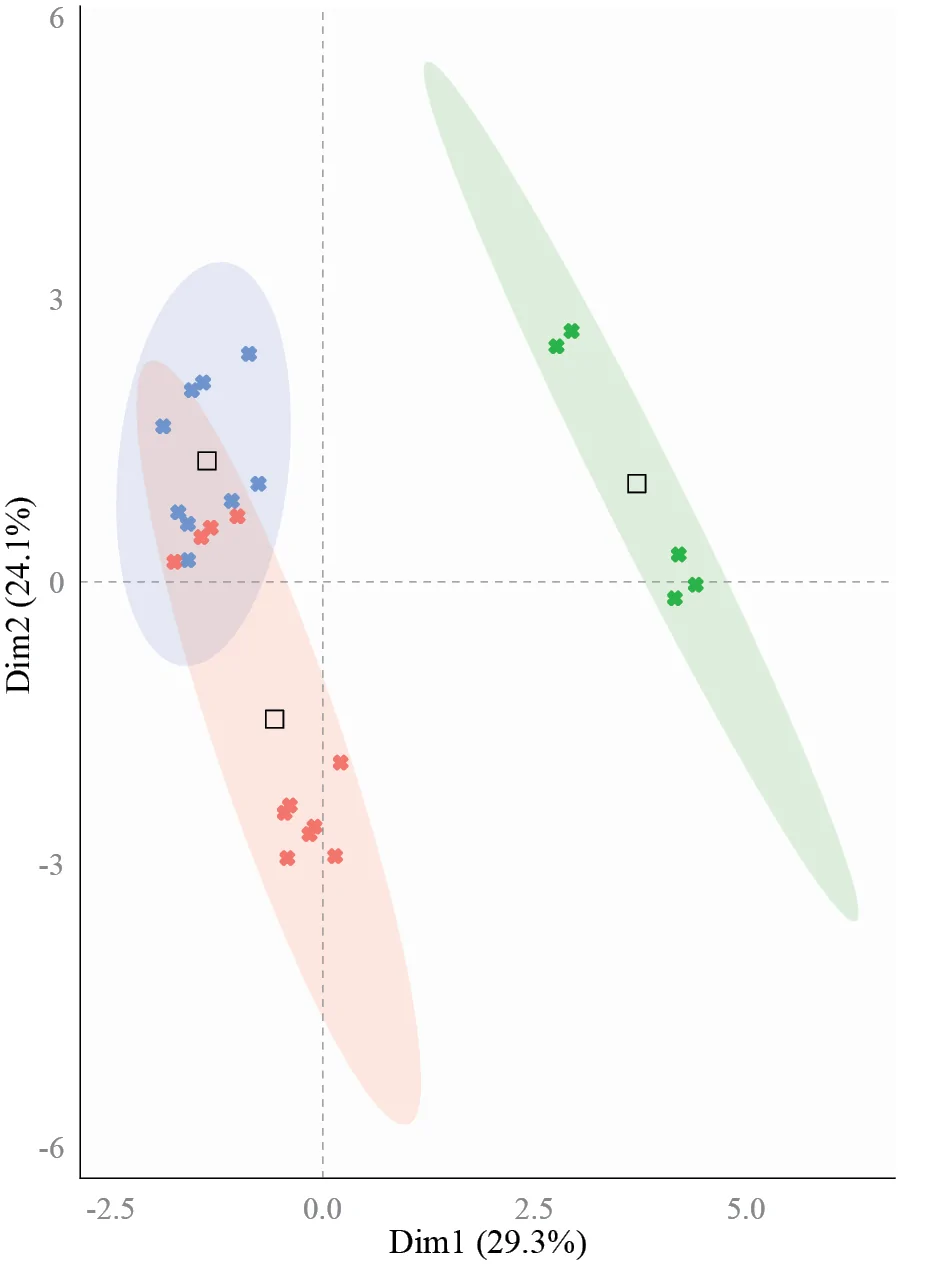
MFA ordination of *Diploderma gulaense* sp. nov. (red), *D. yangi* (blue), and *D. laeviventre* (green) based on size-corrected morphometric, meristic, and categorical data.

The MFA ordination, integrating morphometric, meristic, and categorical characters, recovered three distinct clusters corresponding to the three sampled populations, with the Gula Township population occupying an intermediate position between *D. yangi* and *D. laeviventre*, though closer to *D. yangi* (Fig. 4). The 95% confidence ellipses of the Gula Township population and *D. yangi* partially overlapped, indicating that the two taxa are not completely separated in multivariate morphospace. Pairwise PERMANOVA based on Euclidean distances, however, confirmed highly significant morphological differentiation among all three population pairs (Table 2; all Bonferroni-adjusted p < 0.01). The greatest divergence was recovered between *D. yangi* and *D. laeviventre* (F = 14.28, R^2^ = 0.54, p adjusted < 0.01), whereas the Gula Township population differed from *D. laeviventre* (F = 13.02, R^2^ = 0.48, p adjusted < 0.01) and from *D. yangi* (F = 12.41, R^2^ = 0.41, p adjusted < 0.01). These results place the Gula Township population in a statistically distinct morphological position relative to both recognized species (Grismer 2025). Consequently, the Gula Township population is herein recognized as Diploderma gulaense sp. nov. (described below).

**Table 2.** Pairwise PERMANOVA comparisons based on Euclidean distances among all three species pairs.

**Species pairs**	**F.Model**	**R2**	**p.value**	**p.adjusted**
*Diploderma gulaense* sp. nov. vs *D. yangi*	12.41	0.408	0.00004	0.00012
*D. gulaense* sp. nov. vs *D. laeviventre*	13.02	0.482	0.00034	0.00102
*D. yangi* vs *D. laeviventre*	14.28	0.543	0.00056	0.00168

### Taxonomic account

#### Diploderma gulaense

Taxon classificationAnimaliaBoletalesDiplocystidiaceae

Cai, Yao, Wu & Guo sp. nov.

9697DD8E-C8F4-5D03-9D34-A61F3E9D3E4D

https://zoobank.org/99400B92-00DF-43BA-9244-EF18761BA2CE

Figs 5, 6

##### Type material.

***Holotype***: • CIB123153 (field number CIB-07567), adult male, collected from Gula Township, Zayü County, Xizang Autonomous Region, China (29.0238°N, 97.9546°E, 2402 m a.s.l.) by the team of Zhongyi Yao in August 2019. ***Allotype***: • CIB123154 (field number CIB-CB23CY01), adult female, collected from Gula Township, Zayü County, Xizang Autonomous Region, China (29.0269°N, 97.9519°E, 2406 m a.s.l.) by the team of Boyang Wu in June 2023. ***Paratypes***: • CIB-CB23CY03 (adult female) and • CIB-CB23CY02 (adult male), the same collecting information as the allotype; • CIB-CB23CY16, adult male, from Gula Township, Zayü County, Xizang Autonomous Region, China (29.0067°N, 97.9122°E, 2660 m a.s.l.), collected by the team of Boyang Wu in June 2023; • CIB-07573 (voucher 573), adult male; • CIB-07564 (voucher 564), CIB-07565 (voucher 565), • CIB-07566 (voucher 566), • CIB-07568 (voucher 568), and • CIB-07574 (voucher 574), adult females; • CIB-07569 (voucher 569), juvenile; the same collecting information as the holotype.

**Figure 5.**
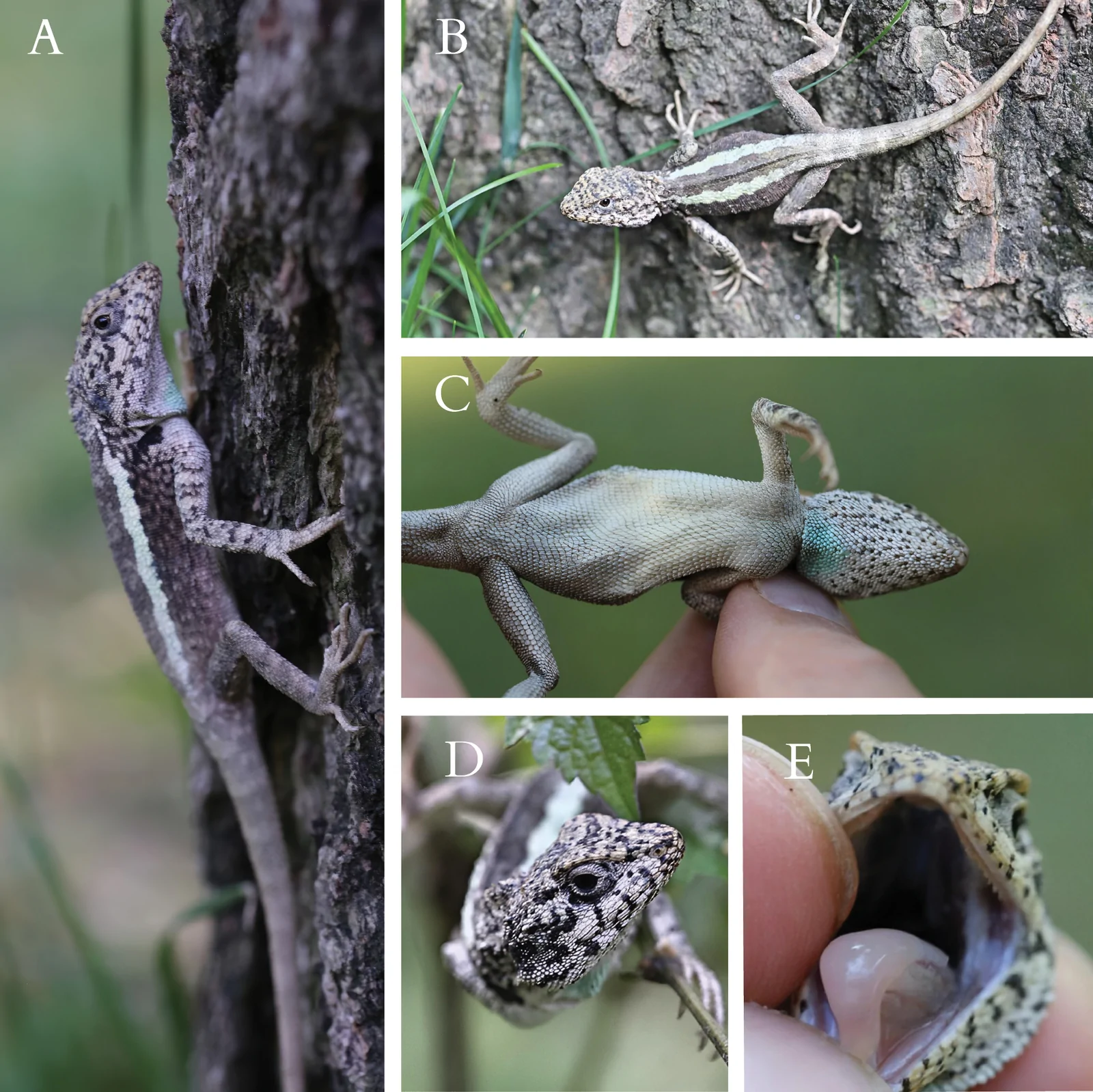
Live coloration of *Diploderma gulaense* sp. nov. (male, CIB-CB23CY17, photographed while healthy). **A**. Lateral view of body; **B**. Dorsal view; **C**. Ventral view; **D**. Lateral view of head; **E**. Oral cavity.

##### Other non-type material.

CIB-CB23CY17, adult male, from Gula Township, Zayü County, Xizang Autonomous Region, China (29.0067°N, 97.9122°E, 2660 m a.s.l.), collected by the team of Boyang Wu in June 2023; however, the specimen is in poor condition as the individual died of illness during temporary captivity.

##### Diagnosis.

*Diploderma gulaense* sp. nov. can be diagnosed from its congeners by a combination of the following morphological characteristics: (1) adult body size moderate, SVL 53.1–63.4 mm in males (*n* = 4), 54.8–70.2 mm in females (*n* = 7); (2) tail relatively long, TAL/SVL 2.07–2.38 (mean 2.18) in males, 1.90–2.26 (mean 2.02) in females; (3) head moderately depressed, HW/HL 0.65–0.75 in males, 0.65–0.74 in females; (4) foreleg length moderate, FLL/SVL 0.46–0.48 in males, 0.43–0.50 in females; (5) hindleg length relatively short, HLL/SVL 0.74–0.80 in males, 0.66–0.77 in females; tympanum concealed by small scales; (6) middorsal crest scales 48–51 in males, 48–54 in females; (7) ventral body scales strongly keeled; (8) transverse gular fold present; (9) skin fold under nuchal and dorsal crests weak; (10) gular spot present, cyan (0, 255, 255) in life; (11) dorsolateral stripes present in males, pale cyan (224, 255, 255), with almost smooth-edged margins; (12) distinct dark radial stripes around eyes; (13) inner-lip coloration smoky-white (245, 245, 245); (14) oral cavity and tongue pale flesh colored (239, 205, 197); (15) ventrolateral body coloration smoky-white to dark grey (169, 169, 169); (16) ventral body uniform smoky-white; (17) hindlimb adpressed forward reaching from anterior margin of tympanum to eye; (18) *D. gulaense* sp. nov. differs statistically in morphospace from *D. yangi* (F = 12.41, R^2^ = 0.408, p = 0.00004, adjusted p = 0.00012) and from *D. laeviventre* (F = 13.02, R^2^ = 0.482, p = 0.00034, adjusted *p* = 0.00102). Separation is driven mainly by gular spot color, dorsolateral stripe coloration, MD, T4S, and size-corrected morphometric variables.

##### Comparisons.

*Diploderma gulaense* sp. nov. is compared with the three other members of the Nu River lineage: *D. laeviventre*, *D. slowinskii*, and *D. yangi*.

From *Diploderma laeviventre*, the new species is distinguished by having a much smaller adult snout–vent length (male SVL 53.1–63.4 mm vs 67–72 mm); a relatively longer tail (TAL/SVL 2.07–2.38 vs 1.97–1.99 in males, 1.90–2.26 vs 1.69–1.84 in females); a relatively longer snout in males (SEL/HL 0.38–0.44 [mean 0.41] vs 0.33–0.35 [mean 0.34]); relatively longer fore- and hindlimbs in males (FLL/SVL 0.46–0.48 vs 0.44–0.46; HLL/SVL 0.74–0.80 vs 0.70–0.74); a lower middorsal crest scale count (MD 48–51 vs 57–59 in males, and 48–54 vs 58–59 in females); strongly keeled ventral body scales (vs smooth or weakly keeled); a different coloration of dorsolateral stripes in males (pale cyan vs cream color); a cyan gular spot (vs pale chrome orange).

From *Diploderma slowinskii*, the new species differs markedly by having a much smaller size (male SVL 53.1–63.4 mm vs 81–98.3 mm; female SVL 54.8–70.2 mm vs 74.8–91.7 mm); a shorter head length (15.3–22.4 vs 24.4–32.9 in males, and 16.1–22.0 vs 23.6–28.5 in females); a relatively shorter tail (TAL/SVL 1.90–2.26 vs 2.37–2.55 in females); a relatively shorter forelimb in males (FLL/SVL 0.46–0.48 vs 0.50–0.56); a different coloration of dorsolateral stripes in males (pale cyan vs pale emerald green to pale pistachio); the presence of a distinct gular spot (cyan in males and females vs absent in both sexes); having concealed tympana (vs exposed).

From *Diploderma yangi*, the new species differs by having a higher gular scale count in females (31–37 vs 25–30); a different coloration of dorsolateral stripes (pale cyan vs pale emerald-green to pale pistachio); a different gular spot coloration (cyan vs pale emerald-green to pale pistachio); no enlarged conical scales immediately behind the rictus (posterior corner of mouth) vs present.

*Diploderma gulaense* sp. nov. differs from *D. brevipes* (Gressitt, 1936), *D. chapaense* (Bourret, 1937), *D. fasciatum* (Mertens, 1926), *D. hamptoni* (Smith, 1935), *D. luei* (Ota, Chen & Shang, 1998), *D. makii* (Ota, 1989), *D. menghaiense* Liu, Hou, Wang, Ananjeva & Rao, 2020, *D. micangshanense* (Song, 1987), *D. nangunhe* Liu, Li, Yang, Hou, Rao & Ananjeva, 2024, *D. ngoclinense* (Ananjeva, Orlov & Nguyen, 2017), *D. polygonatum* Hallowell, 1861, *D. swinhonis* (Günther, 1864), and *D. yunnanense* (Anderson, 1878) by the presence of a transverse gular fold (vs absence).

*Diploderma gulaense* sp. nov. differs from *D. bifluviale* Liu, Wu, Zhang, Yang, Liu, Chen, Chang, Xie & Cai, 2025, *D. daduense* Cai, Liu & Chang, 2024, *D. danbaense* Liu, Hou, Ananjeva & Rao, 2023, *D. drukdaypo* (Wang, Ren, Jiang, Zou, Wu, Che & Siler, 2019), *D. dymondi*, *D. flaviceps* (Wang, Ren, Jiang, Zou, Wu, Che & Siler, 2019), *D. panlong*, *D. qiaojiaense* Liu, Hou & Rao, 2024, *D. shuoquense* Liu, Hou, Rao & Ananjeva, 2022, *D. splendidum* (Barbour & Dunn, 1919), *D. swild*, *D. varcoae*, and *D. vela* (Wang, Jiang & Che, 2015) by the presence of a cyan gular spot in life (vs gular spot being absent); differs from *D. drukdaypo*, *D. flaviceps*, *D. vela*, *D. panchi* Wang, Zheng, Xie, Che & Siler, 2020 and *D. kangdingense* Cai, Zhang, Li, Du, Xie, Hou, Zhou & Jiang, 2022 by having gular spots in both sexes; differs from *D. angustelinea* Wang, Ren, Wu, Che & Siler, 2020, *D. bowoense* Wang, Gao, Wu, Siler & Che, 2021, *D. brevicauda* (Manthey, Denzer, Hou & Wang, 2012), *D. chapaense*, *D. donglangense* Liu, Hou, Ananjeva & Rao, 2023, *D. formosgulae* Wang, Gao, Wu, Dong, Shi, Qi, Siler & Che, 2021, *D. nangunhe*, *D. panchi*, *D. qilin* Wang, Ren, Che & Siler, 2020, and *D. yunnanense* by the presence of a cyan gular spot in life (vs yellow, orange, pinkish flesh, and other warm-toned gular spots); differs from *D. aorun* Wang, Jiang, Zheng, Xie, Che & Siler, 2020, *D. iadinum* (Wang, Jiang, Siler & Che, 2016), *D. xinlongense* Cai, Zhang, Li, Du, Xie, Hou, Zhou & Jiang, 2022, *D. yulongense* (Manthey, Denzer, Hou & Wang, 2012), *D. yongshengense* Liu, Hou, Rao & Ananjeva, 2022, *D. zhaoermii* (Gao & Hou, 2002) by the presence of a cyan gular spot in life (vs yellowish green, green, and blue spots).

*Diploderma gulaense* sp. nov. differs from *D. batangense* (Li, Deng, Wu & Wang, 2001), *D. daochengense* Cai, Zhang, Li, Du, Xie, Hou, Zhou & Jiang, 2022, *D. flavilabre* Wang, Che & Siler, 2020, *D. jiulongense* Liu, Hou, Ananjeva & Rao, 2023, *D. limingense* Liu, Hou, Rao & Ananjeva, 2022, *D. tachengense* Liu, Hou, Ananjeva & Rao, 2023 by the presence of pale cyan dorsolateral stripes in life in males (vs yellow dorsolateral stripes).

*Diploderma gulaense* sp. nov. differs from *D. dymondi* (Boulenger, 1906), *D. panlong* Wang, Che & Siler, 2020, *D. swild* Wang, Wu, Jiang, Chen, Miao, Siler & Che, 2019, and *D. varcoae* (Boulenger, 1918) by having concealed tympana (vs exposed).

##### Description of holotype

**Figure 6.**
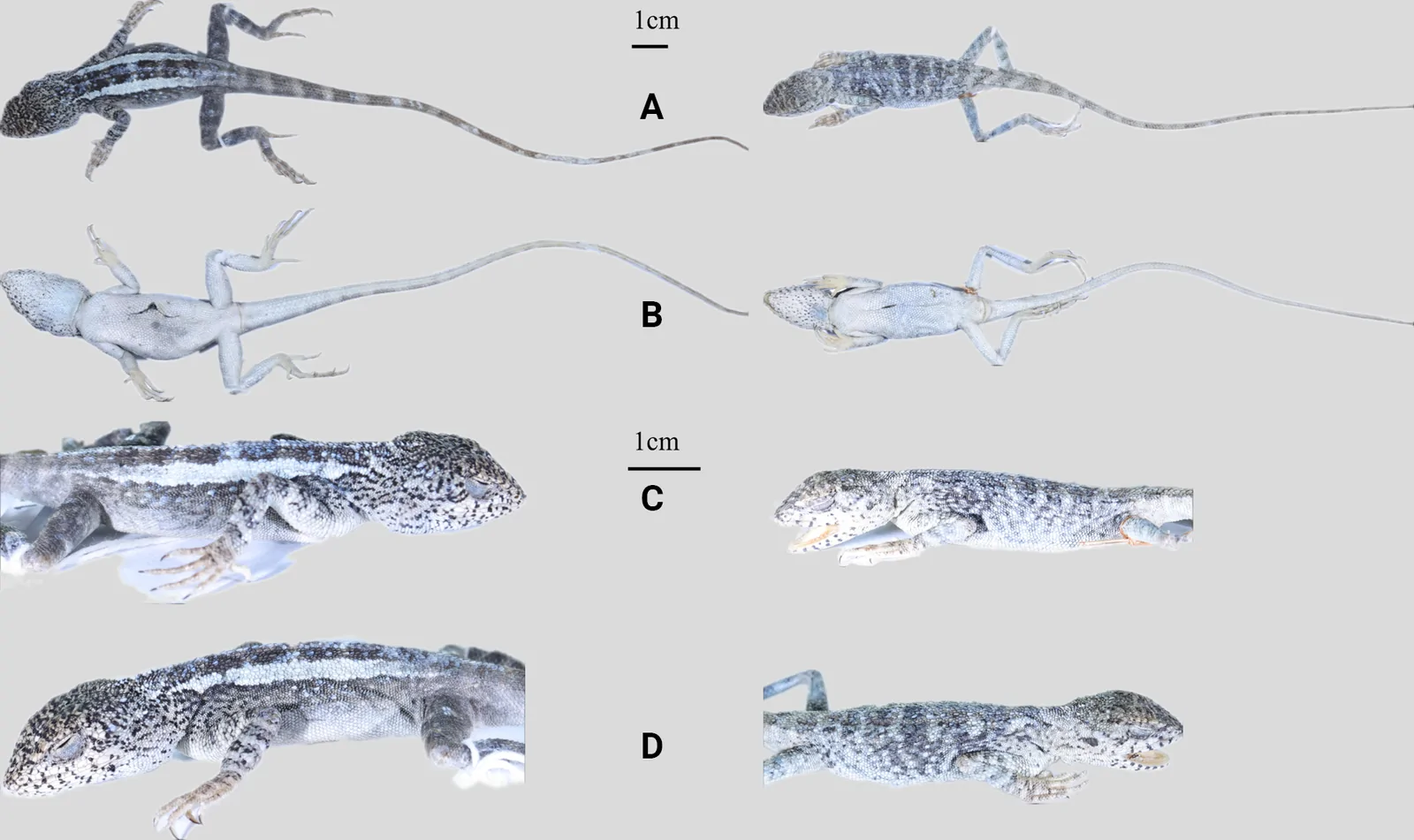
Holotype (left, CIB123153) and allotype (right, CIB123154) of *Diploderma gulaense* sp. nov. **A**. Dorsal view; **B**. Ventral view; **C**. Left lateral view; **D**. Right lateral view.

**(Fig. 6, Table 3)**. An adult male, snout–vent length 63.4 mm; tail length 150.7 mm, tail complete. Head length 22.4 mm, head width 16.9 mm, head depth 11.3 mm; HW/HL 0.75, HD/HL 0.50, HD/HW 0.67. Snout–eye length 8.6 mm, SEL/HL 0.38. Trunk length 26.3 mm, TRL/SVL 0.42. Foreleg length 30.2 mm, FLL/SVL 0.48; hindleg length 48.9 mm, HLL/SVL 0.77; toe IV length 11.4 mm. Length of tallest nuchal crest 0.7 mm, TNC/HL 0.03. Supralabial scale count 8/8, infralabial scale count 8/8. Canthus rostralis scales 11/11, forming a distinct ridge. Nasal separated from first supralabial by a single row of small scales. Suborbital scale rows 3/3. Supratemporals 2/3. Middorsal crest scales 50; nuchal crest weakly erected, skin fold under nuchal crest present but weak, skin fold under dorsal crest present. Gular scales 34, ventral scales 69, both strongly keeled. Finger IV subdigital lamellae 19/19, toe IV subdigital lamellae 26/26. Post-tympanic conical scales 1/1, post-occipital scales 2/2; no enlarged conical scales immediately behind the rictus (posterior corner of mouth). Tympanum concealed by small scales. Transverse gular fold present. Hindlimb adpressed forward reaching between eye and tympanum. Shoulder fold present.

**Table 3.** Morphometric measurements and pholidosis counts of adult *Diploderma gulaense* sp. nov. All morphometric measurements are in the unit of mm, and three individuals (CIB-07564, CIB-07566, CIB-07568) have incomplete tails (indicated by “+”).

**Voucher numbers**	**CIB123153**	**CIB-CB23CY02**	**CIB-07573**	**CIB-CB23CY16**	**CIB123154**	**CIB-CB23CY03**	**CIB-07564**	**CIB-07565**	**CIB-07566**	**CIB-07568**	**CIB-07574**
**Type status**	**Holotype**	**Paratype**	**Paratype**	**Paratype**	**Allotype**	**Paratype**	**Paratype**	**Paratype**	**Paratype**	**Paratype**	**Paratype**
**Sex**	**Male**	**Male**	**Male**	**Male**	**Female**	**Female**	**Female**	**Female**	**Female**	**Female**	**Female**
**Svl**	63.4	60.7	57.9	53.1	66.1	54.8	55.9	70.2	59.4	63.4	66.5
**Tal**	150.7	130.4	123.2	110.1	133.2	104.3	97.3+	158.3	60.5+	84.2+	127.7
**Hw**	16.9	11.2	12.9	10.2	13.0	10.5	13.4	16.3	14.1	13.9	14.0
**Hl**	22.4	17.3	18.3	15.3	18.3	16.1	18.6	22.0	19.7	20.7	20.7
**Hd**	11.3	9.8	8.6	9.3	10.0	8.2	9.2	11.8	10.1	10.1	10.8
**Sel**	8.6	7.6	7.8	6.2	7.9	7.2	7.0	8.0	7.4	6.8	8.4
**Tnc**	0.7	0.5	0.7	0.2	0.6	0.5	0.3	0.7	0.3	0.3	0.4
**Fll**	30.2	29.4	26.6	25.2	30.9	26.2	28.1	32.0	27.9	29.5	28.7
**Hll**	48.9	46.9	42.8	42.5	49.1	40.2	42.4	50.6	46.0	42.1	45.3
**T4l**	11.4	11.5	11.5	11.2	11.7	9.3	8.9	11.4	8.5	10.2	11.4
**Trl**	26.3	31.3	25.2	27.8	32.2	30.2	25.8	30.9	27.8	27.8	32.3
**Sl**	8/8	8/7	10/8	7/7	9/9	8/9	8/8	9/9	8/8	10/10	8/8
**Il**	8/8	8/7	10/9	8/8	9/9	8/8	9/10	9/9	8/8	10/10	9/10
**Cr**	11/11	11/10	11/11	11/11	11/11	10/10	11/11	11/11	11/12	11/11	11/11
**Nsl**	1/1	1/1	1/1	1/1	1/1	1/1	1/1	1/1	1/1	1/1	1/1
**Sor**	3/3	3/3	3/3	2/2	3/3	3/3	4/4	3/3	3/3	4/4	3/3
**Md**	50	50	48	51	54	49	48	49	54	49	48
**Gu**	34	34	33	30	34	34	37	31	35	37	35
**Vn**	69	62	58	60	63	58	61	66	74	70	71
**F4s**	19	17	20	16	17	16	19	19	18	20	17
**T4s**	26	22	23	22	22	21	25	24	25	26	25

##### Coloration of the holotype in alcohol.

Dorsal ground color of head, body, and limbs brownish grey (87, 80, 68). Dorsal head with three indistinct dark brown (92,69,61) transverse stripes. A distinct dark brown radial pattern surrounds the eye; the area between the orbit and supralabials is smoky-white with dark brown radial streaks; supralabials and infralabials similarly marked. Dorsolateral stripes present on each side of body, smoky-white, with almost smooth upper and lower margins, extending from neck to hindlimb insertion. A pale cyan gular spot present on the throat. Ventrolateral body coloration grades from smoky-white to dark grey (169, 169, 169); ventral body uniform smoky-white; palms similarly smoky-white. Dorsal surface of body with six dark brown transverse blotches; limbs with dark brown crossbars on both fore- and hindlimbs. Tail with 18 distinct dark brown transverse bands.

##### Coloration in life of CIB-CB23CY17

**(photographed while healthy) (Fig. 5)**. An adult male, dorsal ground color of head, body, and limbs brownish-grey. Dorsal head with three indistinct dark brown transverse stripes. A distinct dark grey radial pattern surrounds the eye; the area between the orbit and supralabials is smoky-white with dark grey radial streaks; supralabials and infralabials similarly marked. Inner-lip coloration smoky-white. Oral cavity and tongue pale flesh in color. Dorsolateral stripes present on each side of body, pale cyan, with almost smooth upper and lower margins, extending from neck to hindlimb insertion. A distinct cyan gular spot present on the throat. Ventrolateral body coloration grades from smoky-white to dark grey; ventral body uniform smoky-white; palms similarly smoky-white.

##### Variation.

Morphometric and meristic data for the type series of adult *Diploderma gulaense* sp. nov. are summarized in Table 3. Sexual dimorphism is evident in body size and relative tail length. Adult males (n = 4) have a snout–vent length of 53.1–63.4 mm (mean 58.8 mm), whereas adult females (n = 7) are slightly larger, ranging 54.8–70.2 mm (mean 62.3 mm). The tail is relatively longer in males (TAL/SVL 2.07–2.38, mean 2.18) than in females (1.90–2.26, mean 2.02). No significant differences between sexes were observed in other body proportions or pholidosis.

Within males, one paratype (CIB-CB23CY16) has a slightly shorter hindlimb adpressed forward between eye and tympanum (vs reaching the eye in others). Within females, three individuals (CIB-07564, CIB-07566, CIB-07568) have incomplete tails (indicated by “+” in Table 3); these were excluded from TAL ratio calculations. Ventral scale counts vary from 58 to 74, and middorsal crest scales from 48 to 54, with no apparent geographic or sexual clustering. Dorsolateral stripes in males are consistently present and almost smooth-edged; in females they lack typical dorsolateral stripes. The gular spot is cyan in all adults of both sexes.

##### Distribution and habitat.

**Figure 7.**
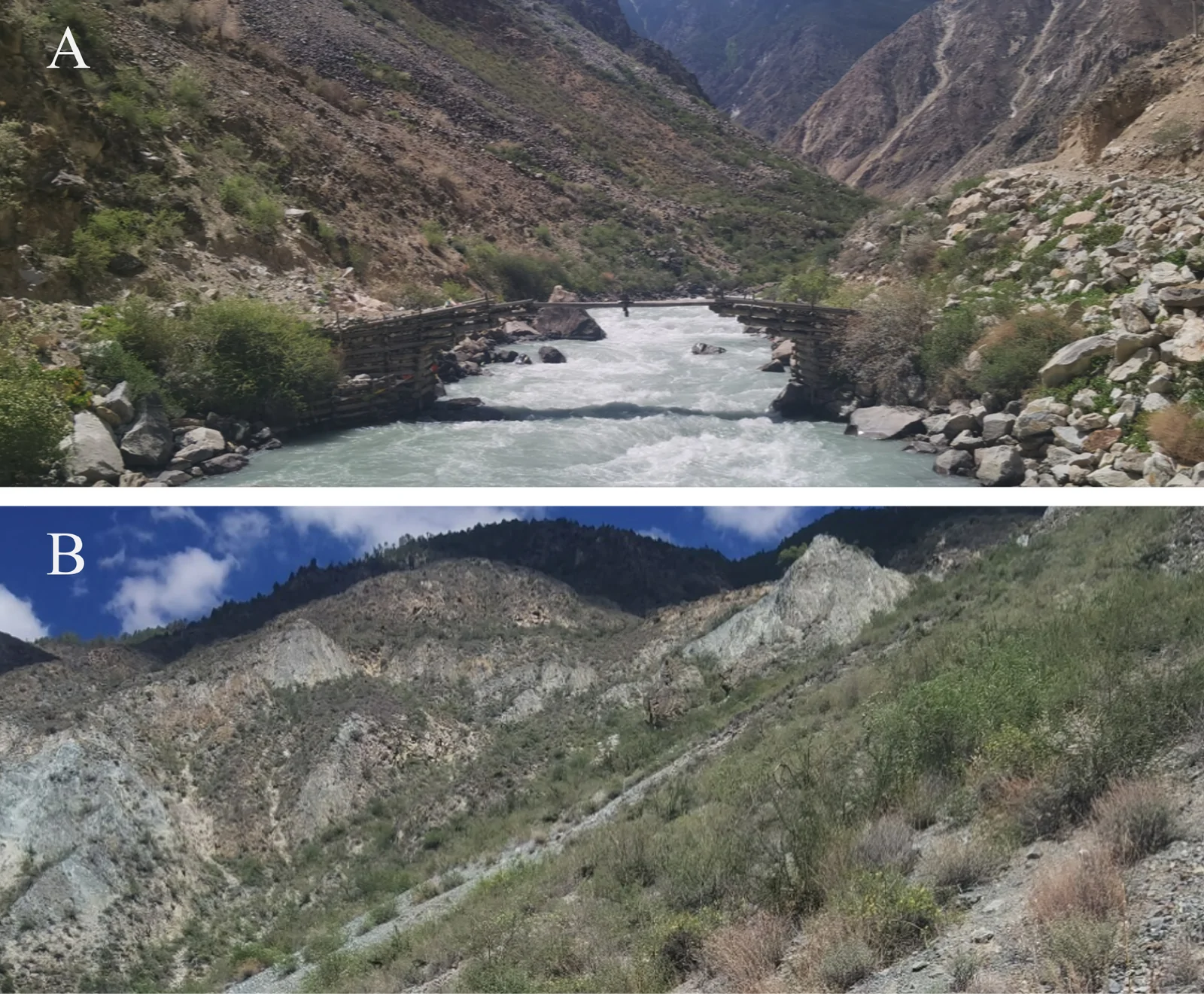
Habitat of *Diploderma gulaense* sp. nov. **A**. Ranbuqu valley; **B**. Microhabitat.

*Diploderma gulaense* sp. nov. is currently known only from the Ranbuqu valley in Gula Township, Zayü County, Xizang Autonomous Region, China. Boyang Wu’s surveys in the area documented the species from Anba Village upstream to Rituo Village, at elevations between 2,332 and 3,300 m. The Ranbuqu River is a first-order right-bank tributary of the middle Nu River. It originates from the Boshula Mountain Range and flows southeastwards through Gula Township before joining the main channel of the Nu River, within a dry-warm valley of the arid river valley system. During these surveys, all individuals were collected from shrubland and rocky areas adjacent to the river, in June and August (Fig. 7).

##### Etymology.

The Latin specific epithet *gulaense* is derived from the Gula Township, where the new species was discovered. The genus *Diploderma* is Greek neuter, and the suffix -*ense* of this new species name is the Latin neuter combinatorial suffix denoting ‘pertaining to’ or ‘originating in’. And we suggest Gula Mountain Lizard as its English common name and 古拉攀蜥 (Chinese phonetic alphabet: gǔ lā pān xī) as its Chinese common name.

## Discussion

Phylogenetic analyses based on mitochondrial ND2 gene sequences recovered the population from Gula Township as a distinct evolutionary lineage and placed it as the sister species of *Diploderma laeviventre*. Together, *D. laeviventre*, *D. gulaense* sp. nov., *D. yangi*, and *D. slowinskii* form a well-supported clade endemic to the Nu River valley (Wang et al. 2016, 2022; Rao et al. 2017).

The PCA provides a descriptive overview of morphometric variation among the three taxa. In males, *D. gulaense* sp. nov. showed only slight overlap with *D. laeviventre* along PC1 and partial overlap with *D. yangi*, whereas broader overlap among taxa was observed in females. We therefore do not interpret the PCA alone as statistical evidence of species-level morphometric differentiation. Instead, the ordination is presented as a visualization of morphometric variation complementary to the molecular phylogenetic and qualitative morphological evidence.

To evaluate the combined discriminatory power of all morphological character types, including meristic and categorical traits such as color pattern and tuberculation, we performed a multiple factor analysis (MFA) following Grismer (2025). The MFA ordination recovered three distinct clusters in morphospace, with the Gula Township population occupying an intermediate position between *D. yangi* and *D. laeviventre* (Fig. 4). The 95% confidence ellipses of *D. gulaense* sp. nov. and *D. yangi* partially overlapped, indicating incomplete separation in multivariate morphospace. Nevertheless, pairwise PERMANOVA based on Euclidean distances confirmed highly significant morphological differentiation among all three taxa (all *p* adjusted < 0.01). The strongest separation was recovered between *D. yangi* and *D. laeviventre* (*F* = 14.28, *R*^2^ = 0.543, *p* adjusted < 0.002), while *D. gulaense* sp. nov. differed from its sister species *D. laeviventre* with a moderately large effect size (*F* = 13.02, *R*^2^ = 0.482, *p* adjusted = 0.00102). This pattern is consistent with the phylogenetic placement of *D. gulaense* sp. nov. as the sister lineage of *D. laeviventre*; although *D. gulaense* is morphologically most similar to *D. yangi* among the three, the two sister species are still statistically separable. The MFA thus provides quantitative, statistically defensible support for recognizing the Gula Township population as a distinct species.

In *Diploderma*, color traits, gular spot and dorsolateral stripe coloration are taxonomically informative and often sexually dimorphic (Cai et al. 2022; Liu et al. 2023; Wang et al. 2024). In the new species, the consistently cyan gular spot in both sexes and dorsolateral stripes in males differ markedly from the cream, orange, or yellow tones in related species, supporting their diagnostic utility.

Despite being genetically most closely related to *Diploderma laeviventre*, *D. gulaense* sp. nov. is morphologically more similar to *D. yangi*. Both species share a cool-toned dorsolateral stripe in males, a conspicuous gular spot in both sexes, and strongly keeled ventral scales. This discordance between genetic affinity and phenotypic resemblance is not contradictory; rather, it sheds light on the speciation history within the clade. The common ancestor of the Nu River lineage probably possessed these morphological traits, which were subsequently modified in *D. laeviventre* (smooth ventrals, orange gular spot) and lost in *D. slowinskii*, while retained in *D. gulaense* and *D. yangi*.

Multiple interacting factors are likely to have contributed to the divergence of *Diploderma gulaense* sp. nov. as a distinct lineage. The rugged topography of the Hengduan Mountains, together with a distinct climatic gradient along the Nu River, ranging from the cold-dry middle valley (inhabited by *D. laeviventre*) through the dry-warm middle valley (home to *D. gulaense* and *D. yangi*) to the warmer, moister lower valley (occupied by *D. slowinskii*), may have substantially restricted gene flow among these populations (Liu et al. 2023, 2024, 2025; Wang et al. 2022, 2024). Sexual selection and sex-specific trait differentiation probably also played a role in driving phenotypic divergence. For instance, the presence or absence and the color of the gular spot, a trait implicated in male competition and species recognition, may have been subject to divergent selection under the contrasting microclimatic conditions of different valleys (Li et al. 2025). In combination, geographical barriers, climatic divergence, genetic drift, and sexual selection are thus all plausible drivers that jointly promoted speciation within the Nu River *Diploderma* clade, though the relative contribution of each remains to be evaluated.

Although our phylogenetic inference relies solely on the mitochondrial ND2 gene, *Diploderma gulaense* sp. nov. was recovered as a distinct and strongly supported evolutionary lineage (BPP/UFBS = 1/100) sister to *D. laeviventre*. Morphological comparisons further revealed a suite of diagnostic characters separating the Gula Township population from its closest relatives, including smaller adult body size, strongly keeled ventral scales, a cyan gular spot, and pale cyan dorsolateral stripes in males. The two species are also geographically separated by approximately 100 km along the Nu River drainage, with the intervening area remaining poorly sampled. The uncorrected ND2 p-distance between *D. gulaense* and *D. laeviventre* (3.06%) falls within the range observed among other recognized species pairs in the genus and, while not used as a sole criterion, is consistent with their distinction as independent lineages. Taken together, the molecular phylogenetic and morphological evidence supports the recognition of *D. gulaense* as a distinct species. Nevertheless, additional nuclear genomic data and broader geographic sampling, particularly in the area between the known localities of *D. gulaense* and *D. laeviventre*, would be valuable for further evaluating lineage boundaries and possible historical gene flow.

The discovery of *D. gulaense* sp. nov. refines our understanding of *Diploderma* diversity and distribution in the Hengduan Mountains. It fills a long-standing distribution gap along the Nu River between *D. laeviventre* in the upper reach (Baxoi County) and *D. slowinskii* along with *D. yangi* in the lower reach (Zayü County and Gongshan County) (Wang et al. 2016, 2022; Rao et al. 2017). At present, the new species is known only from the Ranbuqu valley, a dry-warm tributary of the middle Nu River in Zayü County. This restricted distribution is consistent with the hypothesis that valleys with differing microclimates may each harbour endemic *Diploderma* species (Cai et al. 2024), reflecting a broader trend in the Hengduan Mountains where rugged topography and climatic gradients along river valleys promote micro-endemism across multiple reptile lineages, including *Diploderma* (Liu et al. 2024), *Gloydius* (Ren et al. 2025), and *Gekko* (Xu et al. 2025). Our findings highlight the importance of integrating molecular phylogenetics with detailed morphological comparisons and suggest that the complex river-valley systems of the Hengduan Mountains are likely to function as a key promoter of allopatric speciation in this genus. Future surveys should target similar, poorly sampled dry-warm valleys along the Nu River as well as sites between *gulaense* and *laeviventre*, where further unrecognized diversity probably awaits discovery.

The species is currently known only from the Ranbuqu valley in Zayü County, Xizang, at elevations of 2,332–3,300 m and with an estimated area of occupancy of approximately 16 km^2^. Following the IUCN Red List Categories and Criteria (IUCN Standards and Petitions Subcommittee 2024), we provisionally assess *Diploderma gulaense* as Vulnerable (VU B1ab(iii)+B2ab(iii)). Its very restricted distribution and ongoing habitat degradation from hydropower development in the middle Nu valley make this species prone to extinction. Field surveys are urgently needed to confirm its population status and to guide conservation actions.

## Supplementary Material

XML Treatment for Diploderma gulaense

## Additional information

### Artificial Intelligence (AI) use

The authors accept full responsibility for the content of the manuscript, including the disclosure of any use of AI.

No AI tools were used in the preparation of this manuscript.

### Author ORCIDs

B. Cai https://orcid.org/0000-0002-4214-9060

F. Liu https://orcid.org/0009-0002-7280-1416

A. Budian https://orcid.org/0009-0004-9375-5724

Z. Yao https://orcid.org/0000-0001-5942-9782

B. Wu https://orcid.org/0009-0009-0130-7518

J. Jiang https://orcid.org/0000-0002-1051-7797

X. Luo https://orcid.org/0009-0001-6407-9155

K. Guo https://orcid.org/0000-0001-7508-1173

### Data availability

All the data that support the findings of this study are available in the main text; DNA sequences are deposited in GenBank.

## Supplementary materials

Supplementary material 1 Supplementary tables Data type xlsx Explanation note **table SS1**. Character matrix used for multiple factor analysis (MFA) of morphological traits in *Diploderma gulaense* sp. nov., *D. laeviventre* and *D. yangi*. **table SS2**. Uncorrected genetic pairwise distances (p-distances) (%) between *Diploderma* species in this study based on the mitochondrial ND2 gene sequences. **table SS3**. Summary statistics of PCA analyses of males’ morphometric characteristics in selected members. **table S4**. Summary statistics of PCA analyses of females’ morphometric characteristics in selected members. https://binary.pensoft.net/file/1828672This dataset is made available under the Open Database License (http://opendatacommons.org/licenses/odbl/1.0/). The Open Database License (ODbL) is a license agreement intended to allow users to freely share, modify, and use this Dataset while maintaining this same freedom for others, provided that the original source and author(s) are credited. Bo Cai, Fengjing Liu, Ayimo Budian, Zhongyi Yao, Boyang Wu, Jianping Jiang, Xiaobo Luo, Keji Guo
